# Site-specific *O*-Glycosylation on the MUC2 Mucin Protein Inhibits Cleavage by the *Porphyromonas gingivalis* Secreted Cysteine Protease (RgpB)*[Fn FN2]

**DOI:** 10.1074/jbc.M113.459479

**Published:** 2013-04-01

**Authors:** Sjoerd van der Post, Durai B. Subramani, Malin Bäckström, Malin E. V. Johansson, Malene B. Vester-Christensen, Ulla Mandel, Eric P. Bennett, Henrik Clausen, Gunnar Dahlén, Aneta Sroka, Jan Potempa, Gunnar C. Hansson

**Affiliations:** From the Departments of ‡ Medical Biochemistry and; ¶ Oral Microbiology, University of Gothenburg, 405 30 Gothenburg, Sweden,; the § Copenhagen Center for Glycomics, Department of Cellular and Molecular Medicine and Odontology, University of Copenhagen, DK 2200 Copenhagen, Denmark,; the ‖ Department of Microbiology, Faculty of Biochemistry, Biophysics and Biotechnology, Jagiellonian University, 30-387 Krakow, Poland, and; the ** Oral Health and Systemic Diseases Group, University of Louisville School of Dentistry, Louisville, Kentucky 40202

**Keywords:** Bacterial Pathogenesis, Glycoprotein Biosynthesis, Glycosyltransferases, Mucins, Mucus

## Abstract

The colonic epithelial surface is protected by an inner mucus layer that the commensal microflora cannot penetrate. We previously demonstrated that *Entamoeba histolytica* secretes a protease capable of dissolving this layer that is required for parasite penetration. Here, we asked whether there are bacteria that can secrete similar proteases. We screened bacterial culture supernatants for such activity using recombinant fragments of the MUC2 mucin, the major structural component, and the only gel-forming mucin in the colonic mucus. MUC2 has two central heavily *O*-glycosylated mucin domains that are protease-resistant and has cysteine-rich N and C termini responsible for polymerization. Culture supernatants of *Porphyromonas gingivalis*, a bacterium that secretes proteases responsible for periodontitis, cleaved the MUC2 C-terminal region, whereas the N-terminal region was unaffected. The active enzyme was isolated and identified as Arg-gingipain B (RgpB). Two cleavage sites were localized to IR↓TT and NR↓QA. IR↓TT cleavage will disrupt the MUC2 polymers. Because this site has two potential *O*-glycosylation sites, we tested whether recombinant GalNAc-transferases (GalNAc-Ts) could glycosylate a synthetic peptide covering the IRTT sequence. Only GalNAc-T3 was able to glycosylate the second Thr in IRTT, rendering the sequence resistant to cleavage by RgpB. Furthermore, when GalNAc-T3 was expressed in CHO cells expressing the MUC2 C terminus, the second threonine was glycosylated, and the protein became resistant to RgpB cleavage. These findings suggest that bacteria can produce proteases capable of dissolving the inner protective mucus layer by specific cleavages in the MUC2 mucin and that this cleavage can be modulated by site-specific *O*-glycosylation.

## Teaser

**Background:** MUC2 polymers form the mucus layer of colon that separates luminal bacteria from the epithelium.

**Results:** *P. gingivalis* secrets a protease that cleaves the MUC2 mucin, a cleavage modulated by *O*-glycosylation.

**Conclusion:** Bacteria can disrupt the MUC2 polymer via proteolytic cleavage. However, *O*-glycosylation can inhibit this process.

**Significance:** Bacteria can dissolve the protective inner mucus layer, potentially triggering colitis.

## Introduction

The mucus that covers the epithelium is important for the protection of surface lining epithelia in the gastrointestinal tract. Recent studies have shown that there are two mucus layers in the colon: an outer “loose” layer and an inner mucus layer that is “firmly” attached to the epithelium (1). Goblet cells secrete the MUC2 mucin polymers, which after volume expansion is self-assembled into layered sheets that build the inner mucus layer (2, 3). The thickness of the inner attached mucus layer ranges between 50 and 100 μm (depending on species) and is gradually converted to the outer layer (1, 3). Both the inner and the outer mucus layers are primarily composed of the MUC2 mucin, a large glycoprotein whose central regions consists of PTS domains rich in the amino acids proline, threonine, and serine that after *O*-glycosylation result in the typical mucin domain. The N- and C-terminal domains are less glycosylated, are stabilized by numerous disulfide bonds, and make the MUC2 C-terminally dimeric and N-terminally trimeric (4, 5). Because the MUC2 monomer is ∼2.5 MDa in mass, this polymerization generates enormous net-like structures (3, 6). The MUC2 polymer normally remains intact in the inner mucus layer and expands to form the outer mucus layer (6).

The human colon has a high number of commensal bacteria, some of which live in symbiosis with their host (7). The number of microorganisms in the adult intestine is estimated to be 10^13^–10^14^, a number well exceeding the number of cells in the host body (8). The microbial community is diverse and complex and comprises approximately l,000 bacterial species (9). The majority of these organisms belong to the phyla *Bacteroidetes* and *Firmicutes* families and are found in the large intestine where they live in the outer loose mucus layer (2). Interestingly, the inner mucus layer normally acts as a barrier that does not allow bacteria to reach the epithelial cells and thus limits the direct contact between the host and the bacteria. However, when there is no mucus layer, as in the Muc2-null mice, the direct contact between bacteria and epithelium triggers severe inflammation (1). The pathogenic bacterium *Citrobacter rodentium* has specific mechanisms to colonize the firm mucus layer in mice causing colitis (10). *O*-Glycosylation of serine or threonine with GalNAc, often called mucin-type glycosylation, is mediated by a family of 20 polypeptide *N*-acetylgalactosaminyltransferases (GalNAc-Ts)^3^ (11). These GalNAc-Ts have distinct but partly overlapping acceptor substrate specificities and are differentially expressed in cells and tissues, which provides for differential and dynamic regulation of *O*-glycosylation of proteins and the specific sites of *O*-glycan attachment on proteins. Specific GalNAc-Ts either initiate *O*-glycosylation or complete attachment of *O*-glycans in protein regions with dense *O*-glycosylation such as those found on mucins (11). Several GalNAc-Ts are linked to diseases, and the molecular mechanism for deficiency of one GalNAc-T, GalNAc-T3, has been clarified and shown to cause the disease familial tumoral calcinosis caused by a lack of site-specific *O*-glycosylation of a single Thr residue in a proprotein convertase inactivation site of the factor fibroblast growth factor FGF23 (12, 13). Further studies have demonstrated more general roles of site-specific *O*-glycosylation adjacent to sites of regulated proteolytic protein processing (14). *Entamoeba histolytica* is a parasite that is able to invade the host mucosa and pass over the inner mucus layer. *E. histolytica* was shown to secrete a cysteine protease that cleaves the MUC2 protein core at a specific site between the second mucin domain and its disulfide bond-stabilized C terminus (15). This cleavage disrupts the MUC2 polymer and allows the amoeba to penetrate the inner mucus layer. We then asked whether there are bacteria in the colon that also can cleave the MUC2. We initially analyzed the typical commensal bacteria *Lactobacillus brevis*, *Lactobacillus plantarum*, *Lactobacillus bulgaricus*, and *Bifidobacterium lactis* for secreted proteases that could cleave and dissolve the MUC2 mucin protein core, but none of these showed any proteolytic activity (16). This is in line with the bacteria having their habitat in the lumen and outer mucus layer and lacking mechanisms of penetrating and utilizing the inner mucus layer. In search of other bacteria that can be found in the colonic flora and that could potentially produce MUC2 specific proteases, we tested whether *Porphyromonas gingivalis* was such a candidate. *P. gingivalis* belongs to the phylum *Bacteroidetes* and is a nonmotile, Gram-negative, rod-shaped, anaerobic bacterial pathogen. The bacterium can be found in colon but is more abundant in the oral cavity, where it is implicated in the pathogenesis of periodontitis because of its ability to deter antibacterial attack by the immune system, and triggers a chronic inflammatory reaction via secretion of a family of cysteine proteases called gingipains (17). We now demonstrate that the *P. gingivalis* Arg-gingipain B (RgpB) is able to cleave the MUC2 mucin at a specific site that will cause the mucus polymer to dissolve. Furthermore, we show that this cleavage site can be selectively blocked by site-specific *O*-glycosylation of the Thr located adjacent to the cleavage site by GalNAc-T3.

## EXPERIMENTAL PROCEDURES

### 

#### 

##### Bacteria and Culture Conditions

*P. gingivalis* W83 (18) obtained from CCUG (Culture Collection University of Gothenburg, Gothenburg, Sweden) and mutants ΔKgp, ΔRgpA, and ΔRgpA/RgpB were plated on Brucella agar (BBL Microbiology Systems) enriched with 5% defibrinated horse blood, 0.5% hemolyzed blood, and 5 μg/ml menadione and incubated overnight at 37 °C in anaerobic jars with 95% H_2_ and 5% CO_2_. Selected colonies were cultured anaerobically in basal medium-PY-peptone yeast extract (1.0 g of peptone, 1.0 g of yeast extract, 0.4 ml of resazurin solution, 4 ml of salt solution, 100 ml of distilled water, and 0.05 g of cysteine HCl-H_2_O) for 96 h at 30 °C. The bacteria were pelleted by centrifugation at 4,000 rpm for 30 min, and the supernatants were carefully removed and used in the subsequent studies.

##### Antibodies and Proteins

The MUC2N3 and MUC2C2 polyclonal antisera were generated against the MUC2 peptides CPKDRPIYEEDLKK and CIILRPDNQHVILKPGDFK, respectively, as described previously (4, 5). Goat anti-rabbit immunoglobulin coupled to alkaline phosphatase (DAKO) was used as secondary antibody for all immunoblotting. The design and production of the recombinant MUC2 N and C termini, consisting of residues 1–1792 and 4198–5179, respectively, of the human MUC2 protein N terminus flanked by GFP and the Myc tag, are described in detail elsewhere (5, 19). Purified gingipain RgpB from the *P. gingivalis* strain HG66 was produced as previously described (20, 21).

##### MUC2 Proteolytic Cleavage by P. gingivalis Secretions and Purified RgpB

All of the protease assays were performed as follows. Secretions from *P. gingivalis* or 200 ng of RgpB were preincubated in Dulbecco's PBS in the presence or absence of the cysteine specific protease inhibitor E-64 (100 μm) (Roche Applied Science) at 37 °C for 30 min, followed by the addition of 1 μg of purified recombinant MUC2 N or C terminus and overnight incubation at 37 °C. Controls were incubated in Dulbecco's PBS without the addition of *P. gingivalis* secreted material or RgpB. The digestion was terminated by the addition of 2× SDS-PAGE sample buffer (4% SDS, 125 mm Tris-HCl, pH 6.8, 30% (v/v) glycerol, 5% (v/v) bromphenol blue, supplemented with 200 mm DTT for reducing conditions) and heated at 95 °C for 5 min. The samples were directly analyzed by SDS-PAGE using precision protein standards (Bio-Rad) as markers. Western blotting was performed as described (15). After transfer and blocking the blots were incubated with anti-MUC2 N3 or C2 antibodies and developed using nitroblue tetrazolium/5-bromo-4-chloro-3-indolyl phosphate (Promega).

##### Fractionation of the Secretions from P. gingivalis

*P. gingivalis* cultured supernatant was prefiltered (0.45-μm mini capsule; Pall) and dialyzed (Spectra/Por Dialysis Membrane, molecular weight cutoff of 6,000–8,000; Spectrum Laboratories) three times against 20 mm Tris-HCl, pH 8.0. The dialyzed medium was fractionated by ion exchange chromatography (MonoQ HR 5/5; GE Healthcare). The samples were loaded in buffer A (20 mm Tris-HCl, pH 8.0) and eluted using a linear 45-min gradient toward 100% buffer B (1 m NaCl in 20 mm Tris-HCl, pH 8.0), and the fractions were collected at 2-min intervals. The protease activity in each individual fraction was determined by incubation with MUC2-C followed by SDS-PAGE and immunoblotting using the anti-MUC2C2 antibody. Aliquots of the active fractions were analyzed by SDS-PAGE under reducing conditions and stained using Imperial protein stain (Thermo) for subsequent protein identification by mass spectrometry.

##### In-gel Digestion, Mass Spectrometry, and Data Analysis

Bands of interest were excised from the SDS-PAGE gels and washed for 20 min with 25 mm NH_4_HCO_3_ in 50% CH_3_CN. The gel pieces were dried by vacuum centrifugation, reconstituted in 20 μl of 10 mm DTT in 25 mm NH_4_HCO_3_, and incubated for 1 h at 56 °C. The remaining solution was removed, 20 μl of 55 mm iodoacetamide in 25 mm NH_4_HCO_3_ was added, and the samples were left for 30 min at room temperature. The gel bands were washed twice more, dried, and reconstituted in 7 μl of trypsin or endoproteinase Lys-C (10 ng/μl) (Promega) in 25 mm NH_4_HCO_3_ and incubated at 37 °C overnight. The digestion reaction was quenched by the addition of 25 μl of 50% CH_3_CN, 1% CH_3_COOH, and the peptides were extracted twice. Pooled supernatants were dried and resolved in 15 μl of 0.1% formic acid prior MS analysis. The samples were analyzed by nLC-ESI MS/MS (LTQ Orbitrap XL ETD; Thermo), and 2 μl of the sample was injected and separated online as described earlier (22). After loading in 0.2% formic acid, the peptides were separated using a 15- or 35-min 10–50% gradient toward acetonitrile. The shorter gradient was used for analyses of the 29-mer peptide, whereas for cleavage site determination and protein identification, the longer gradient was applied. The mass spectrometry analysis was performed in a data-dependent mode with automatic switching between scan modes, performing MS/MS on the top six most intense ions per precursor scan. MS scans in the mass range of *m*/*z* 400–2,000 were obtained in the Orbitrap and MS/MS fragmentation scans in the LTQ ion trap using collision-induced dissociation. Selected sequenced ions were dynamically excluded for 30 s. Raw spectral data were converted using extract_msn.exe (version 3.2 Thermo), identified by MASCOT (version 2.2.04 Matrix Science) and searched against the SwissProt protein database (release 57.12) with the following parameters: (i) one missed cleavage Trypsin or LysC; (ii) tolerance 5 ppm (precursor), 0.5 Da (fragment ions); (iii) charge state 2+, 3+, 4+; (iv) carbamidomethyl cysteine (fixed), oxidized methionine (variable); and (v) taxonomy other bacteria (41,537 entries). Additional parameters were included for the cleavage site determination: a variable TMPP modification on the peptide N terminus, semitryptic cleavage, and searched against human (20,242 entries) supplemented with our in-house MUCIN database available at our website containing the recombinant MUC2 C-terminal sequences. Protein identifications were considered if based on at least two unique peptide identifications with an ion score of >20.

##### Identification of GalNAc-Ts Expressed in Human Colonic Epithelia by Mass Spectrometry

Two sigmoid biopsies were obtained from patients referred to routine colonoscopy in which the colonic mucosa appeared macroscopically normal. Approval was granted by the Human Research Ethical Committee of the University of Gothenburg, and written informed consent was obtained from all study subjects. Epithelial cells were isolated as described previously (23) with slight modifications. Briefly, the tissues were washed in PBS for 5 min followed by addition of PBS containing 3 mm EDTA and 1 mm DTT and incubated at 4 °C for 60 min while being gently shaken. The solution was replaced with fresh PBS, and epithelial cells were dissociated from the tissue by vigorous shaking for 30 s. The remaining tissue was removed from solution, and the cells were pelleted by centrifugation at 500 rpm, resolved in 500 μl of 2 m NaCl, 1 mm EDTA in 10 mm HEPES, pH 7.4, and lysed by tip probe sonication. The membrane proteins were extracted as described (24), with all centrifugation steps performed at 100,000 × *g* for 20 min. The proteins were solubilized in 0.1 m DTT, 4% SDS in 0.1 m Tris-HCl, pH 7.6, and digested using trypsin (Promega) according to the filter-aided sample preparation method (25) on a 10,000-kDa cutoff filter (NanoSep; Pall). Recovered peptides were fractionated using ZIC-HILIC (3.5 μm; SeQuant, Sweden) packed in a fused silica capillary (150 mm × 0.32 mm inner diameter) with 5 mm ammonium acetate in 0.5% formic acid, 95% acetonitrile as mobile phase A and 5 mm ammonium acetate as mobile phase B. Elution was performed with a gradient between 0 and 50%; six fractions were collected, dried under vacuum, and reconstituted in 15 μl of 0.1% C_2_HF_3_O_2_. Both mass spectrometry and spectral data analysis were performed as described.

##### TMPP Labeling of Proteolytically Cleaved MUC2 C Terminus

For determination of the proteolytic cleavage site, the α-amine groups of MUC2-C were specifically labeled using (*N*-succinimidyloxycarbonyl-methyl)tris(2,4,6-trimethoxyphenyl) phosphonium bromide (TMPP) after incubation with either supernatants from *P. gingivalis*, RgpB or PBS. The pH of the samples was first titrated to 8.2 by the addition of 0.1 m Na_2_HPO_4_, after which 5 μl of freshly prepared 20 mm TMPP reagent (Sigma) resolved in 70% EtOH was added. The reaction was quenched after 1 h by the addition of 1 μl of 0.1% hydroxylamine, and the samples were analyzed by SDS-PAGE under reducing conditions and stained using Imperial protein stain (Thermo) followed by in-gel tryptic digestion and mass spectrometry analysis.

##### Edman Sequencing of the Major MUC2 C-terminal Cleavage Products

Recombinant MUC2-C (10 μg) was incubated with 5 μg of the secreted material from *P. gingivalis* or 200 ng of RgpB at 37 °C overnight. The samples were separated by SDS-PAGE, blotted to PVDF membrane, and stained with Imperial protein stain (Thermo). Bands of interest were excised and sequenced by Edman degradation on a Procise 492 protein sequencer (Applied Biosystems).

##### Inhibition of RgpB-mediated Proteolysis of MUC2 by Site-specific O-Glycosylation

The effect of GalNAc *O*-glycosylation on RgpB cleavage of MUC2 was determined by *in vitro* assays using the synthetic peptide AWTPTPTPLSTPSIIRTTGLRPYPSSVLI (hMUC2, amino acid 4306–4335) (Schafer-N) covering the identified cleavage site IR↓TT. The peptide was glycosylated using recombinant human GalNAc-Ts as described earlier (26) using either GalNAc-T1, -T2, -T3, -T4, -T5, -T7, or -T12. Glycopeptides were subsequently glycosylated by T7 or T12. The sites of GalNAc incorporation were characterized by mass spectrometry (LTQ-Orbitrap XL ETD; Thermo) after 30 min of digestion with trypsin using direct syringe infusion (2 μl/min). Fragmentation spectra were generated using electron transfer dissociation with fragment ion detection in the Orbitrap. For inhibition studies, the glycosylated peptides were diluted in PBS to a concentration of 1 μg/μl, and 1 μl was incubated for 5 min at 37 °C with 1 μl of *P. gingivalis* secretion, RgpB (10 ng/μl) or 1 μl of *E. histolytica* secretion with the addition of 8 μl of PBS. The nonmodified peptide was treated under identical conditions. The reaction was quenched by addition of 100 μl of 0.2% formic acid and directly analyzed by nLC-ESI MS/MS using a 15-min gradient. The data were interpreted manually using Xcalibur software (version 2.1; Thermo).

##### Immunohistochemistry on Colon Sections

Sections were cut from frozen blocks of five case patients with normal appearing mucosa adjacent to colon adenocarcinomas at a thickness of 5 μm and mounted on gelatin-coated slides. Every fifth section was stained by hematoxylin-eosin and used as reference during evaluation. The sections were fixed in 10% cold buffered neutral formalin for 15 min and incubated with anti-GalNAc-T3 mAb UH5 (2D10) at 4 °C overnight (27). Bound mAbs were detected with FITC-conjugated rabbit anti-mouse immunoglobulin absorbed with human serum (F-261; Dako Denmark). In one case, a double-layer immunofluorescence technique was used. The first layer was mixed with a polyclonal antibody against human MUC2 non-*O*-glycosylated TR 1:500, and after blocking with goat serum (1:10), the second layer was replaced with a mixture of FITC-conjugated goat anti-mouse immunoglobulin (115-545-166; Jackson ImmunoResearch, Baltimore, MD) (1:200) and goat anti-rabbit immunoglobulin 115−295-144 (Jackson) (1:200) and mounted with Vectashield containing DAPI (Vector Laboratories, Burlingame, CA). Fluorescence microscopy was performed using a Zeiss Axioskop 2 plus, and confocal images were obtained with an LSM780 (Carl Zeiss).

##### Generation of CHO K1 Cells Coexpressing MUC2-C and GalNAc-T3

A CHO K1 clone stably expressing SMG-MUC2C (5) was cultured in Iscove's modified Dulbecco's medium + 10% fetal bovine serum (Lonza, Verviers, Belgium) and transfected with pcDNA3.1zeo/GalNAc-T3 using Lipofectamine 2000 (Invitrogen). Stable clones were selected in the presence of both 250 μg/ml Geneticin (Invitrogen) and 250 μg/ml Zeocin (Invitrogen). Spent culture supernatant without serum from one clone expressing high levels of GalNAc-T3 was collected and used in the RgpB cleavage assay as described above. 20 ng of RgpB was added to 2 μg of purified MUC2-C and incubated for 5, 15, or 30 min at 37 °C before analysis by nonreducing SDS-PAGE as described.

## RESULTS

### 

#### 

##### Degradation of the MUC2 C-terminal Region by P. gingivalis Secreted Cysteine Proteases

**FIGURE 1.**
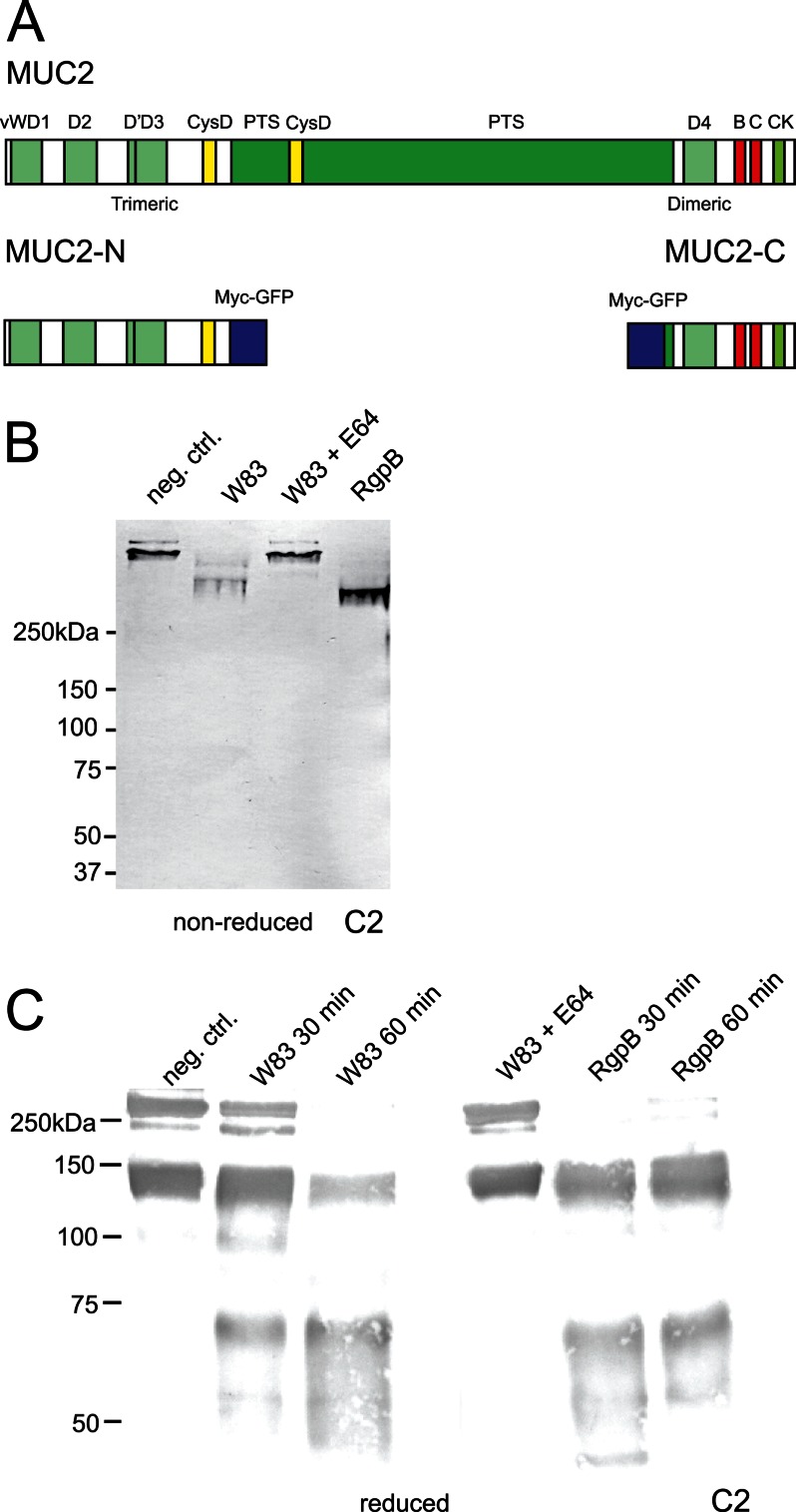
**The C terminus of the MUC2 mucin can be degraded by cysteine proteases secreted by *P. gingivalis*.** *A*, schematic presentation of the domain structure of the MUC2 mucin and recombinant fusion proteins. *B* and *C*, gel electrophoresis and immunoblot analysis of the MUC2 C terminus incubated with *P. gingivalis* secreted material. The MUC2-C was incubated overnight with secretions from *P. gingivalis* W83 with or without preincubation with E-64, purified protease RgpB, or buffer alone. *B*, the samples were analyzed under nonreducing conditions by Western blotting with anti-MUC2C2. The W83 secretion-treated bands show a decrease in molecular mass compared with the control and E-64 inhibited. *C*, analyses under reducing revealed two distinct bands at 130 and 65 kDa; identical results were obtained using the purified enzyme RgpB. *neg. ctrl.*, negative control.

We have previously shown that some typical commensal bacteria do not have any proteolytic activity on the MUC2 mucin (16). However, we screened some potentially more aggressive bacteria for MUC2 degradation and found that *P. gingivalis* was such a candidate. To specifically analyze this, we earlier generated the recombinant trimeric MUC2-N and dimeric MUC2-C proteins that contained the N- or C-terminal cysteine-rich parts (N terminus is 1396 amino acids and C terminus 981) of the MUC2 as outlined in Fig. 1*A* (5, 19). Cultured supernatants from bacteria were incubated with the recombinant MUC2-N or MUC2-C, and the cleaved products were analyzed by immunoblotting using either anti-MUC2N3 or anti-MUC2C2 antisera. These studies showed that secretions of *P. gingivalis* did not affect the MUC2 N terminus (data not shown) but degraded the MUC2-C dimer. This observation was investigated further.

MUC2-C incubated with the secreted material from the *P. gingivalis* strain W83 was analyzed by SDS-PAGE under nonreducing conditions and immunoblotted using the anti-MUC2C2 antiserum. Because the MUC2-C is a large dimer with an estimated mass of 470 kDa (19), it migrated slowly, whereas the cleaved product migrated further into the gel, indicating the presence of secreted proteases capable of digesting MUC2-C (Fig. 1*B*). When MUC2-C was instead analyzed under reducing conditions, it migrated as a monomer at ∼250 kDa (Fig. 1*C*). When the cleaved products were analyzed under reducing conditions two bands were seen, one at 65 kDa and one at ∼130 kDa (Fig. 1*C*). Preincubating the secretions with E-64, the diagnostic inhibitor of papain-like cysteine proteases, could abolish this proteolytic cleavage. E-64 reversibly inhibits arginine-gingipains (RgpA and RgpB) but has no effect on Kgp activity (21). This suggests that cysteine proteases other than Kgp are involved in MUC2-C cleavage. The lower band observed in the control sample at 150 kDa is due to the autocatalytic degradation of the GDPH sequence (29).

##### Identification of the Cysteine Proteases in P. gingivalis Secretions Responsible for Cleavage of the MUC2 C Terminus

**FIGURE 2.**
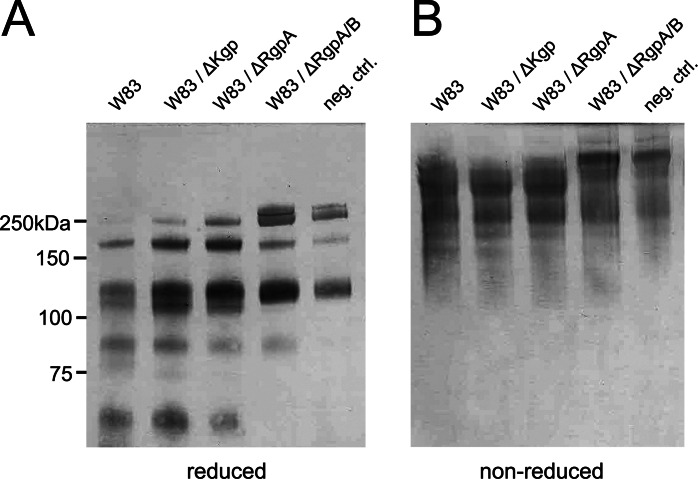
**Immunoblot analysis of *P. gingivalis* secretions from different gingipain mutant strains.** MUC2-C was incubated overnight with secretions from three different mutants of *P. gingivalis* and subjected to immunoblotting using anti-MUC2C2. The immunoblots show that under both reducing (*A*) and nonreducing (*B*) conditions, Kgp and RgpA mutants show identical band patterns, compared with the wild type (W83) where a distinct band at 65 kDa indicates the presence of the protease. The double RgpA/RgpB mutant shows identical results as the control, indicating that RgpB is the protease involved in MUC2-C degradation. *neg. ctrl.*, negative control.

To determine the nature of the protease that cleaved the MUC2, the secreted products were separated by ion exchange chromatography, and the fractions were tested for proteolytic activity on the MUC2-C (supplemental Fig. S1). The products were analyzed by SDS-PAGE and stained with the anti-MUC2C2 antiserum. Fractions 8–12 showed proteolytic activity indicated by loss of the full length and the appearance of the band at 65 kDa representing the major cleavage fragment. Active *P. gingivalis* fractions were analyzed by SDS-PAGE, and stained bands were excised for subsequent analysis by mass spectrometry. Protein database searches identified various *P. gingivalis* proteins, of which three were known proteases (supplemental Table S1). The proteases RgpA, RgpB (Arg-gingipain A and B), and KgP (Lys-gingipain) were identified in all active fractions. All three are gingipains and thus cysteine proteases, confirming the initial protease inhibition results (Fig. 1*C*). RgpA and RgpB belong to the gingipain family of broad specific proteases and cleave at the P1 position of Arg (30), whereas the gingipain Kgp is a Lys-specific protease (31). To identify which gingipain was involved in degradation of the MUC2-C, secretions from *P. gingivalis* mutant strains lacking one or two of the proteases (ΔKgp, ΔRgpA, and ΔRgpA/RgpB) were incubated with MUC2-C and analyzed under reducing and nonreducing conditions by immunoblotting using the anti-MUC2C2 antiserum (Fig. 2). The wild type strain W83 and mutants for ΔKgp and ΔRgpA degraded the protein as observed by the appearance of the 65-kDa fragment or loss of full-length MUC2-C under reducing and nonreducing conditions, respectively (Fig. 2). Proteolytic activity was observed in the two mutants ΔKgp and ΔRgpA, suggesting that the protease responsible for cleavage was RgpB. The double mutant ΔRgpA/RgpB further confirmed this because the cleavage was completely abolished. RgpB has a broad pH optimum in the range of pH 9.5 (100% activity) down to pH 5 (50%), which covers the pH range of the colon (20).

To further demonstrate that the RgpB is able to cleave MUC2 in the same way as the culture supernatants, purified RgpB was incubated with MUC2-C, and the products were analyzed by SDS-PAGE under nonreduced and reduced conditions (Fig. 1, *B* and *C*). The results show the same 65-kDa fragment appeared, thus confirming that the *P. gingivalis* gingipain RgpB is able to cleave the MUC2 mucin in its C-terminal end.

##### Characterization of the MUC2 C-terminal Cleavage Products

**FIGURE 3.**
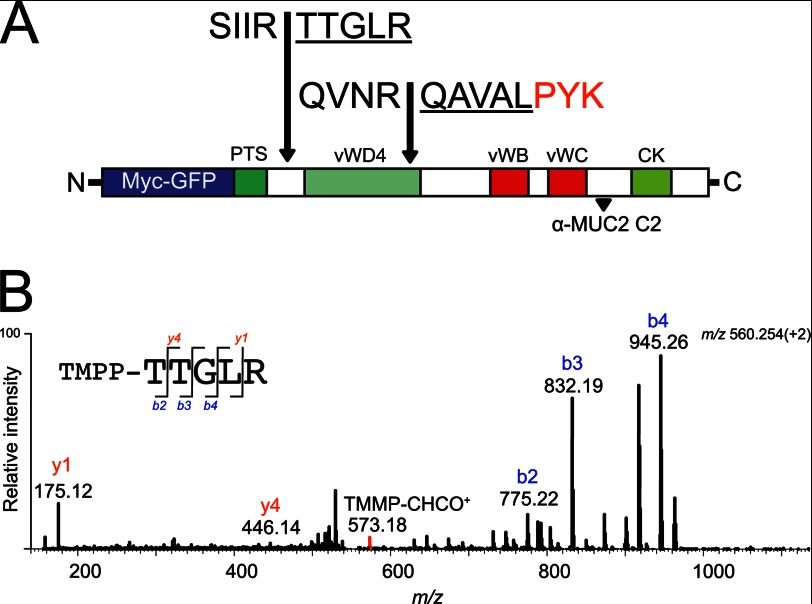
**Neo N-terminal sequences after proteolytic cleavage as determined by both Edman degradation and mass spectrometry.** *A*, schematic representation of the human MUC2-C corresponding to the region directly after the PTS domain (amino acids 4198–5179) flanked at the N-terminal side by GFP and the Myc tag. The two cleavage sites identified by Edman sequencing at ↓TTGLR and ↓QAVAL are highlighted, plus the peptide TTGLR and QAVALPYK as confirmed by mass spectrometry analysis. The localization of the epitope for the anti-MUC2C2 polyclonal antibody is highlighted at the far C terminus. *B*, fragmentation spectra of the TMPP-labeled peptide TTGLR. The MUC2 C terminus was incubated with *P. gingivalis* secretions and labeled using TMPP specifically derivatizing the newly formed N terminus. Mass spectrometry analysis identified the peptide TTGLR labeled at its N terminus, indicating proteolytic cleavage at this site. The TMPP-CHCO+ reporter ion is indicated in *red*, b-ions are in *blue*, and y-ions are in *red*.

Degradation of MUC2 after incubation with the *P. gingivalis* supernatant or RgpB suggested specific cleavage sites in MUC2. To analyze this aspect, the *P. gingivalis* culture supernatant was incubated with MUC2-C, the products were separated by SDS-PAGE, and the bands were excised and analyzed by Edman sequencing. The results of the 130-kDa band showed an N terminus with the sequence ^4322^TTGLR, and the 65-kDa band showed an N terminus with the sequence ^4566^QAVAL (Fig. 3*A*), both with arginine at position P1.

To confirm the cleavage, an α-amine specific labeling technique was applied prior to mass spectrometry analyzes. The TMPP labeling technique ensures that the peptide remains in its ionic state required for sequencing and increases its overall hydrophobicity, which separates it from most peptides during reverse phase chromatography (32, 33). The RgpB-treated MUC2-C product was TMPP-labeled prior to gel separation by SDS-PAGE and stained with Coomassie, and both the 130- and 65-kDa bands were in-gel digested using trypsin. Extracted peptides were analyzed by nLC-ESI MS/MS, and the spectral data were searched for semitryptic peptides. The TMPP-modified peptide TTGLR eluting at 36.2 min with an *m*/*z* of 560.25 (2+) was identified as the neo N terminus of the MUC2 protein at amino acid residue 4322 in the 130-kDa band (Fig. 3*B*). The identified peptide was labeled at its N terminus, and the fragmentation spectra revealed the distinct reporter ion TMPP-CHCO^+^ at *m*/*z* 573.18 (1+) and a predominant b-ion series as induced by the TMPP labeling (32). The modified peptide could not be identified in the control MUC2 C-terminal band. The N-terminal peptide in the 65-kDa band could not be identified potentially because of its increased hydrophobicity induced by the TMPP label. However, protein digestion using the lysine specific enzyme Lys-C identified the peptide QAVALPYK at *m*/*z* 445.26 (2+) corresponding to the N terminus in the 65-kDa band. Thus, both cleavage sites were identified by mass spectrometry and Edman sequencing.

RgpB therefore cleaves the MUC2 mucin at two positions. The MUC2-C has a high number of Cys amino acids that form multiple disulfide bonds, rendering this part of the protein surprisingly protease-resistant. The ^4566^QAVAL cleavage is within such a region. On the other hand, the ^4322^TTGLR cleavage is located N-terminally prior to the first Cys of the MUC2 C terminus after the large mucin domain. Cleavage at IR↓TT is therefore expected to cause a separation of the two products as shown for *E. histolytica* (15), whereas when cleaved at the NR↓QA, the protein is still held together via disulfide bonds. This interpretation is consistent with the relative small shift in size when analyzed on nonreducing gels where the C-terminal part of MUC2-C is still held together as a dimer. As for *E. histolytica*, the *P. gingivalis* cleavage at the IR↓TT is therefore expected to disrupt the MUC2 polymer network.

##### O-Glycosylation of MUC2 Adjacent to the IR↓TT Cleavage Site

Because the RgpB cleavage site has two adjacent Thr residues that could potentially be *O*-glycosylated, we determined whether these sites could be glycosylated *in vitro*, whether glycosylation would block RgpB cleavage, and whether the required GalNAc-Ts were expressed in the colon. The repertoire of GalNAc-Ts expressed in human colon is not fully elucidated (11). To characterize which isoforms are expressed in the colon, we first used proteomics analysis on epithelial cells isolated from human colonic biopsies. Analysis of the membrane proteome identified expression of seven GalNAc-Ts (T1, T2, T3, T4, T5, T7, and T12) (Table 1).

**TABLE 1:** **GalNAc transferases identified by mass spectrometry in human sigmoid colonic epithelium**

Accession number	Gene	Unique peptides identified	Sequence coverage
			%
Q10472	*GALNT1*	2	4.7
Q10471	*GALNT2*	5	8.4
Q14435	*GALNT3*	9	18.8
Q8N4A0	*GALNT4*	8	14.4
Q7Z7M9	*GALNT5*	6	7.6
Q86SF2	*GALNT7*	11	19.3
Q8IXK2	*GALNT12*	7	15.1

**FIGURE 4.**
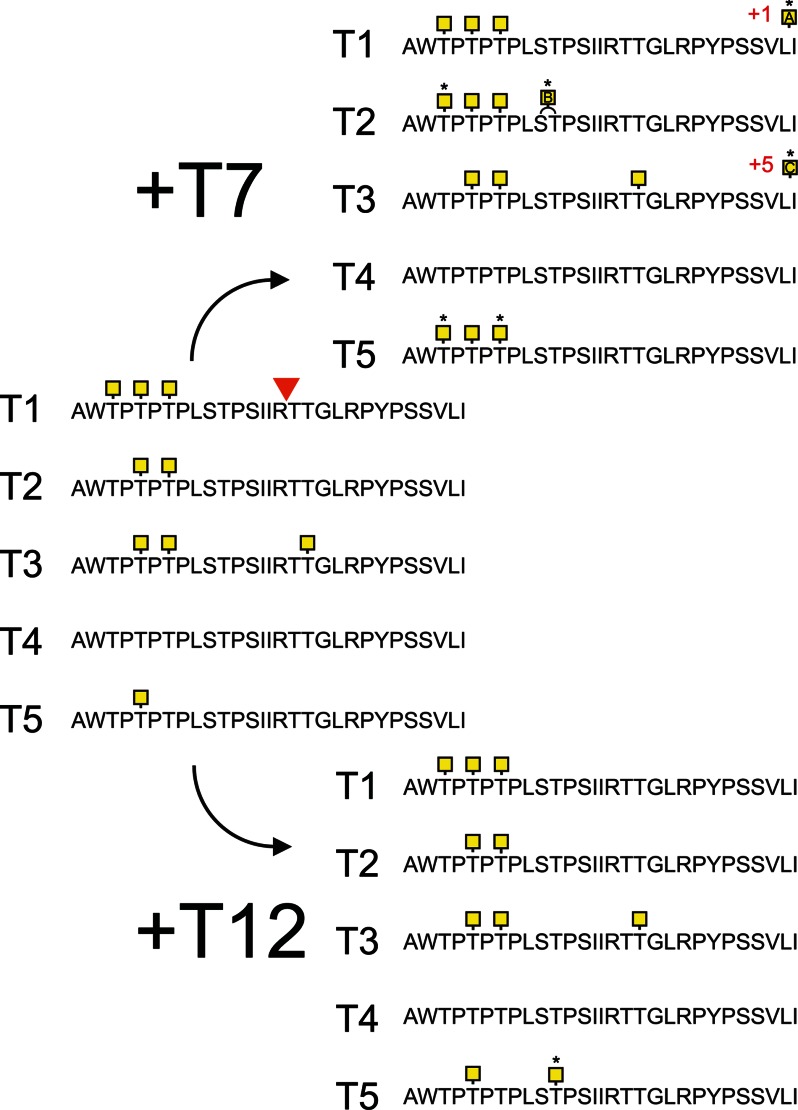
**Analysis of *in vitro O*-glycosylation sites by different GalNAc-T isoforms on a 29-mer MUC2 peptide.** The peptide corresponding to the amino acids 4306–4335 were *O*-glycosylated with the GalNAc-Ts T1, T2, T3, T4, and T5, followed by the glycopeptide specific GalNAc-T7 or -T12. Sites were characterized by mass spectrometry using electron transfer dissociation fragmentation. The sites of GalNAc incorporation are highlighted and marked with *asterisks* when added by T7 or T12. The RgpB cleavage site is indicated in the sequence at the IR TT position. Sites of incorporation could not be assigned for the GalNAc-T1 and -T2 glycosylated peptides (marked *A* and *B* in *yellow squares*). Glycosylation by the combination of GalNAc-T3 with -T7 added on average eight GalNAc residues to the peptide where modified sites could not be unambiguously assigned due to the complexity (marked *C* in a *yellow squares*).

To test whether the ^4320^IR↓TT region can be glycosylated and whether this can inhibit RgpB cleavage, a synthetic 29-mer peptide covering the cleavage region with and containing 10 potential *O*-glycosylation sites (MUC2-pep 1, AWTPTPTPLSTPSIIRTTGLRPYPSSVLI, possible *O*-glycosylation sites underlined) was glycosylated *in vitro* by the recombinant human GalNAc-Ts that were found to be expressed in the colon. GalNAc-T1 added one to four GalNAc residues, where the addition of three GalNAcs was the major product (Fig. 4). GalNAc-T2 incorporated GalNAc residues at Thr^4311^ and Thr^4315^; however, none of these sites were predicted to affect the RgpB cleavage. Glycosylation with GalNAc-T3 showed an incorporation of the highest number of GalNAc residues (up to 6), with the addition of three GalNAc residues comprising the major product (supplemental Fig. S3). Mass spectrometry analysis of the tryptic digest of the GalNAc-T3 glycosylated peptide with the addition of three to five GalNAc residues showed that the Thr^4325^ was occupied at the P2′ position for the RgpB cleavage site (supplemental Fig. S2). GalNAc-T5 added only one residue to the peptide. Supplemental Fig. S3 shows the obtained glycopeptides where the peak intensities indicate the relative amount of each glycoform. The GalNAc-T7 and -T12 enzymes did not add any GalNAc to the naked peptide. However, prior GalNAc modifications on the peptide by GalNAc-T3 allowed GalNAc-T7 to add up to nine additional GalNAc residues to nine of ten potential sites. Interestingly GalNAc-T12, which has been associated with colon cancer (34), only added one or two GalNAcs to the GalNAc-T5 primed peptide (Fig. 4). To summarize, only GalNAc-T3 was able to add a GalNAc to one of the two Thr adjacent to the RgpB cleavage site.

##### Inhibition of the RgpB Cleavage of MUC2-C by Site-specific O-Glycosylation

**FIGURE 5.**
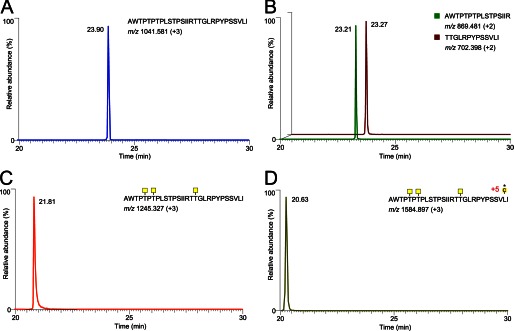
**Extracted ion chromatograms of the mass spectrometry analysis on the 29-mer synthetic peptide modified by GalNAc-T3 and digested with RgpB.** *A*, control incubated for 5 min at 37 °C in PBS; only the noncleaved peptide at *m*/*z* 1041.58 (+3) was observed. *B*, incubation with RgpB resulted in cleavage at the IR4320↓TT position as confirmed by MS/MS sequencing of the two peptides AWTPTPTPLSTPSIIR and TTGLRPYPSSVLI. *C*, extracted ion chromatograms of the GalNAc-T3 glycosylated peptide after 5 min of incubation with RgpB. The peptide was not affected as observed by the peak at *m*/*z* 1245.32 (+3) of the full peptide with three GalNAc residues. *D*, the GalNAc-T3 glycosylated peptide further glycosylated by GalNAc-T7. The eluting peptide peak at *m*/*z* 1584.89 (+3) of the full peptide with eight GalNAc residues was not cleaved by RgpB.

The effect of GalNAc *O*-glycans adjacent to the identified RgpB cleavage site IR↓TT was addressed using the *in vitro* glycosylated peptides. Both glycosylated and nonglycosylated peptides were incubated for 5 min with purified RgpB, after which the reaction was quenched. Mass spectrometry analysis of the control and RgpB-treated nonglycosylated peptide showed a single peak for the intact 29-mer peptide (Fig. 5*A*) and two peptide fragments AWTPTPTPLSTPSIIR and TTGLRPYPSSVLI (Fig. 5*B*) after incubation with the protease. In contrast, incubation of the GalNAc-T3 glycosylated peptide containing three GalNAc residues with the RgpB protease did not result in cleavage, and only the intact glycopeptide was found after incubation (Fig. 5*C*). Glycopeptides produced by any of the other single GalNAc-Ts were all degraded by the RgpB protease. This indicated that selective glycosylation of Thr^4322^ by GalNAc-T3 rendered the glycopeptide resistant to degradation. The combination of GalNAc-T3 and -T7 produced a highly glycosylated peptide with nine of ten sites occupied that was also fully resistant to proteolysis (Fig. 5*D*).

These results suggest that a single GalNAc residue attached to the second Thr (Thr^4322^) is the only modification that is required to render the IRTT sequence completely resistant to RgpB cleavage. The effect of *E. histolytica* secreted material on both the nonmodified and GalNAc-T3-modified peptide was also studied (15). This showed that this site-specific glycosylation also inhibited the secreted *E. histolytica* protease. In the nonmodified peptide, the cleavage site ^4320^RT↓TG was confirmed. In addition, exoprotease activity was observed, resulting in various truncated peptides (supplemental Fig. S4*A*). The GalNAc-T3-modified peptide became resistant to both endo- and exoprotease activities (supplemental Fig. S4*B*).

##### Localization of GalNAc-T3 to Goblet Cells in Human Colon

**FIGURE 6.**
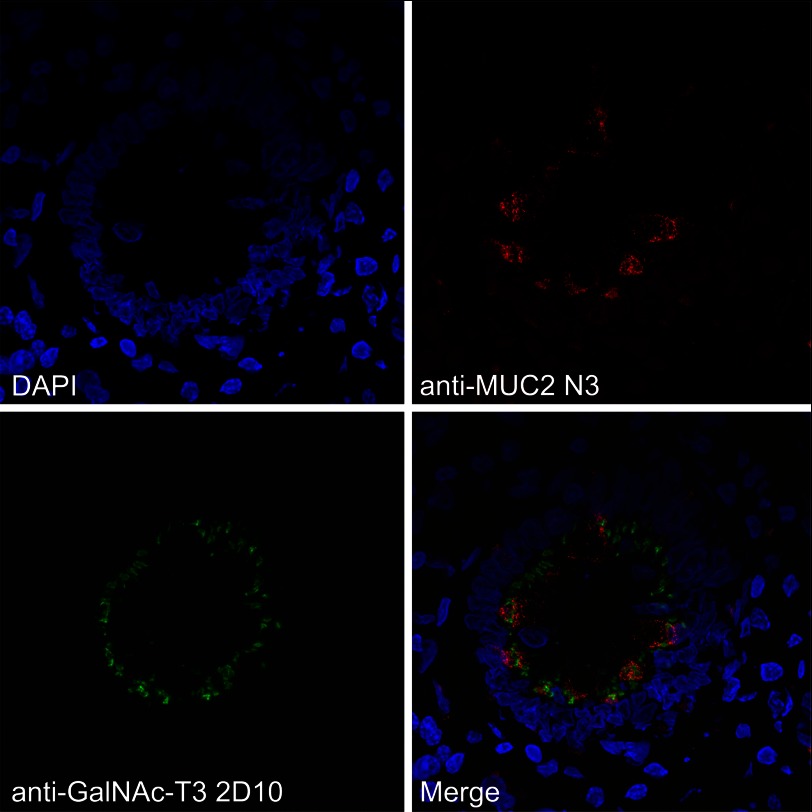
**GalNAc-T3 is colocalized with the MUC2 mucin in human colon goblet cells.** GalNAc-T3 was stained with mAb 2D10, the MUC2 (unglycosylated) was stained with the anti-MUC2TR antiserum, and the nuclei were stained with DAPI.

Because the GalNAc-T3 enzyme was the only GalNAc-T found in the colon epithelium that could modify and block the RgpB cleavage site in MUC2, we confirmed that the transferase was expressed in the Golgi apparatus of colonic goblet cells. Immunohistology of human colon tissue with an anti-GalNAc-T3 mAb revealed staining of both enterocytes and goblet cells (Fig. 6). The colocalization of MUC2 and GalNAc-T3 was further confirmed by staining goblet cells with the antiserum anti-MUC2TR that only recognizes the endoplasmic reticulum-early Golgi non-*O*-glycosylated MUC2 precursor. The colocalization of MUC2 and GalNAc-T3 suggests that MUC2 is *O*-glycosylated at the IRTT sequence, because *in vitro* analysis of GalNAc-T substrate specificities correlate well with *in vivo* glycosylation (11).

##### Expression of GalNAc-T3 in CHO-K1 Cells Secreting MU2C-C Makes the Protein Resistant for RgpB

**FIGURE 7.**
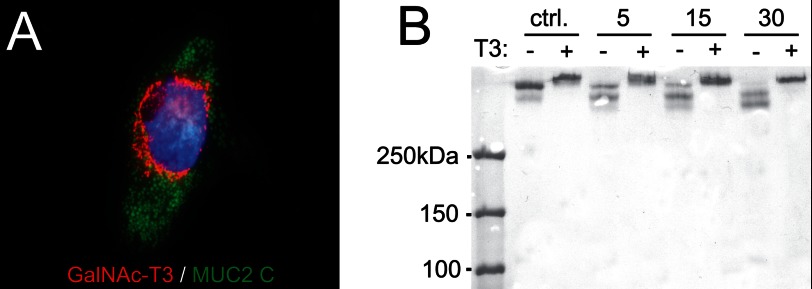
**RgpB cleavage of MUC2 C-terminal construct coexpressed with or without the GalNAc-T3 transferase.** *A*, costaining of the MUC2 C terminus in *green*, GalNAc-T3 transferase in *red*, and the nuclei in *blue*. The Golgi localized fraction of the MUC2 C terminus shows a clear localization with the transferase. *B*, MUC2-C produced in CHO cells with (+) or without (−) GalNAc-T3 was incubated for 5, 15, or 30 min at 37 °C with RgpB. SDS-PAGE analysis visualized by Coomassie staining. MUC2-C expressed in CHO cells without GalNAc-T3 was completely degraded after 30 min as observed by the loss of the upper band, whereas the MUC2-C coexpressed with GalNAc-T3 remained intact. *ctrl.*, control.

The recombinant MUC2-C used in these studies was produced in CHO-K1 cells. Recent transcriptome analysis of this cell line revealed that only GalNAc-T2, -T7, -T11, and -T20 were expressed (35). This observation and the observed RgpB cleavage of MUC2-C suggest that the Thr^4322^ is not glycosylated in the recombinant protein. CHO-K1 cells coexpressing MUC2-C and GalNAc-T3 were generated to determine whether this made MUC2 resistant to cleavage (Fig. 7*A*). The MUC2-C secreted from these cells was incubated for various time points with the recombinant RgpB, and the products were analyzed by nonreducing SDS-PAGE stained by Coomassie (Fig. 7*B*). The MUC2-C from cells expressing GalNAc-T3 showed full resistance to cleavage by RgpB. The results suggest that MUC2-C produced in CHO-K1 cells is not glycosylated at Thr^4322^ and that coexpression with GalNAc-T3 can completely inhibit the degradation of MUC2 by RgpB.

## DISCUSSION

The anaerobic bacterium *P. gingivalis* was found to secrete a protease capable of cleaving the MUC2 mucin at two specific sites in the C-terminal region, one which will cause the MUC2 polymeric network to dissolve. Of the three gingipains secreted by *P. gingivalis*, it was only RgpB that was able to cleave the MUC2 mucin. In contrast, the 1,400-amino acid-long N-terminal part of MUC2 was not affected by RgpB. The central region of MUC2 is ∼2,800 amino acids long and contains two mucin domains that are highly glycosylated. Because of this glycosylation, the mucin domains are highly resistant to proteases and are not expected to be cleaved by proteases (36). The 980-amino acid-long C-terminal part of MUC2 showed two cleavage sites. One is localized to the NR↓QA sequence within the VWD4 domain where the cleavage site is surrounded by numerous cysteines that are involved in disulfide bond formation (Fig. 1*A*). The NR↓QA cleavage does not disrupt the protein as suggested by the analysis of the nonreduced cleavage products. Thus, a cleavage at this site will not disrupt the disulfide bond-stabilized MUC2 polymer. However, the second cleavage site is localized prior to the first cysteine in the MUC2 C-terminal VWD4 domain and is therefore not stabilized via disulfide bonds. Proteolytic cleavage at this site is therefore expected to disrupt the polymeric network of the MUC2 mucin.

The MUC2 mucin is also cleaved by secreted cysteine protease from the colon pathogen *E. histolytica* (15). One of the cleavages by *E. histolytica* protease was within the VWD4 domain at the KT↓TP sequence 817 amino acids further C-terminal to the here identified *P. gingivalis* cleavage site. Both of these *E. histolytica* and *P. gingivalis* cleavages were stabilized with disulfide bonds, and the products did not separate. The other RgpB cleavage site was identified at the IR↓TT sequence. Interestingly, this is only one amino acid before the *E. histolytica* cleavage that occurred between the two Thr in RT↓TG. In this case it was shown that the *E. histolytica* was able to dissolve the colon mucus because it disrupted the covalent structure necessary for an intact MUC2 polymer. Thus, the RgpB will potentially also dissolve the MUC2 mucus gel because the cleavage is at the same location. It is interesting that only two cleavage sites are used by two different pathogenic organisms for degrading MUC2. The cleavage shared area around the IRTT sequence, which causes disruption of the MUC2 polymer, suggests that this region is a weak point in the human MUC2 mucin, a sequence that is absent in the mouse Muc2 (15).

The IR↓TT cleavage site contains two Thr that could be *O*-glycosylated. Testing recombinant GalNAc-Ts on a synthetic peptide revealed that GalNAc-T3 added GalNAc to the second Thr in the IRTT sequence and that this glycosylation inhibited the cleavage by RgpB. None of the other GalNAc-Ts found in the colonic epithelium was able to glycosylate this site. However, combining GalNAc-T3 with GalNAc-T7 further glycosylated the IRTT-containing peptide and rendered it completely resistant to cleavage by RgpB. No other GalNAc-Ts, single or in combination with the glycopeptide specific GalNAc-transferases, affected the IRTT site, and the obtained glycopeptides remained susceptible to RgpB cleavage. When MUC2-C was expressed in CHO cells, it was susceptible to RgpB digestion and only after introduction of GalNAc-T3 MUC2-C became resistant to cleavage by RgpB, confirming the role of GalNAc-T3 in glycosylation of MUC2-C and its protection from proteolysis *ex vivo*. The repertoire of GalNAc-Ts endogenously expressed in CHO-K1 includes GalNAc-T2, -T7, -T11, and -T20 (35), and coexpression of GalNAc-T3 with the endogenous GalNAc-T7 should in accordance with the *in vitro* glycosylation generate a fully resistant MUC2 mucin as observed for the synthetic peptide.

**FIGURE 8.**
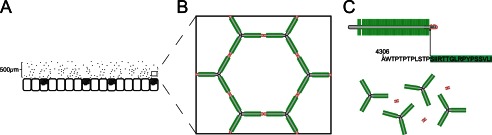
**Schematic model of MUC2 polymer degradation by the protease RgpB.** *A*, the MUC2 mucin forms a dense polymeric network upon secretion by the goblets cells in colon (1, 3). *B*, the MUC2 mucin polymer forms highly organized ring-like structures held together by disulfide linkage at the MUC2 N- and C-terminal regions. *C*, the central part of MUC2 consists of two mucin domains that are protected from proteolytic cleavage by its high glycosylation. Disruption of the mucus polymer at the termini is prevented by internal disulfide bonds. The position of the cleavage site Arg^4320^ identified in this study is found in between these two regions and would result in complete disruption of the mucus gel.

We identified protein expression of GalNAc-T1, -T2, -T3, -T4, -T5, -T7, and -T12 in the colonic epithelial cells. We could also show that the critical GalNAc-T3 is colocalized with MUC2 in the goblet cells, suggesting that the IRTT sequence of MUC2 is glycosylated *in vivo*. GalNAc-T3 deficiency is a rare congenital condition resulting in the disease familial tumoral calcinosis characterized by hyperphosphatemia and ectopic ossifications and caused by a lack of GalNAc-T3-mediated site-specific *O*-glycosylation of FGF23 that protects it from inactivating proprotein convertase processing (12, 13). These patients have not been reported to have any gastrointestinal symptoms. However, this may have been overlooked because of the severe nature of the disease. The *in vivo* functions of individual GalNAc-Ts and their contribution to the *O*-glycoproteome is largely unexplored, as are the final biological consequences of changes in the *O*-glycoproteome as an effect of changes in expression of GalNAc-Ts. We are beginning to gain insights into the *O*-glycoproteome of a single GalNAc-T, and it has been demonstrated to give qualitative changes in the *O*-glycoproteome (38). Given that GalNAc-T3 seems to be ubiquitously expressed in human colon, one would envision that pathogenic bacterial enzymes of the type described here would not be able to degrade the MUC2 mucus layer under normal conditions and have gross effects. However, because *O*-glycosylation site occupancy often varies or is incomplete, this may render subjects more susceptible to mucus degradation by pathogens such as *P. gingivalis* and *E. histolytica* Earlier work by our group has shown that *E. histolytica* secreted proteases are capable of degrading MUC2 produced by LS 174T cells, a cell line that endogenously express GalNAc-T3 (39). Because the MUC2 mucin produced in this cell line was dissolved by the *E. histolytica* secretion, it emphasizes that the glycosylation is often incomplete. It can thus be suggested that also humans, which normally have GalNAc-T3 in their goblet cells, will be variably susceptible to MUC2 cleavage by the *E. histolytica* and *P. gingivalis* proteases. The observation that glycosylation at a single amino acid is important for protecting the protein core of MUC2 from degradation supports the idea that especially *O*-glycosylation is important for intestinal protection. Recently, we demonstrated that deletion of a single glycosyltransferase that extends the *O*-glycans on the C3 of the GalNAc, the Core 1 enzyme, caused spontaneous colitis (40). This finding indicates that glycosylation of proteins, especially the mucins, is very important for its protective properties and has developed in an evolutionary balance between the host and commensal and pathogenic bacteria. *P. gingivalis* is a well known pathogen that causes periodontitis (41). *P. gingivalis* is an anaerobic bacterium and is expected to also thrive in the colon. Although this has not been studied in detail, recent metagenomic studies confirmed that the *Porphyromonadaceae* family and *P. gingivalis* are represented among the colon microbiota (28, 37). That *P. gingivalis* secretes a protease able to cleave MUC2 at a specific site where it will disrupt the MUC2 polymeric network suggests that it can also degrade the inner mucus layer in colon when not fully glycosylated (Fig. 8). The function of this layer is to separate the intestinal microbiota from the epithelial cells, such a bacterium would probably increase the number of bacteria that can reach the epithelium. As we know that increased contact with intestinal bacteria, as exemplified by the mouse lacking the Muc2 mucin (1), causes severe inflammation, it is not impossible that *P. gingivalis* may be one of several factors that could contribute to colitis.

## Footnotes

3 The abbreviations used are:

GalNAc-T: *N*-acetylgalactosaminyltransferases
TMPP: (*N*-succinimidyloxycarbonyl-methyl)tris(2,4,6-trimethoxyphenyl) phosphonium bromide.
